# *HROpen*—open, accessible and now with an impact factor!

**DOI:** 10.1093/hropen/hoac034

**Published:** 2022-07-29

**Authors:** Siladitya Bhattacharya, Edgardo Somigliana, Andrew Williams

**Affiliations:** Editor-in-Chief, Human Reproduction Open; Deputy Editor, Human Reproduction Open; Managing Editor, ESHRE Journals

*Human Reproduction Open* now has an impact factor, its first since its launch 5 years ago. This is welcome news, which demonstrates that the papers published in this journal are visible to, and are being cited by, our peers. The concept of a metric to inform decisions about prioritizing journals for indexing was first introduced by Eugene Garfield, and, since 1975, the impact factor for a journal has been calculated as the number of citations in a particular year over all citable publications in that journal over the two preceding years. Traditionally used as a measure of the quality of a journal, it comes as no surprise, that from the very start, impact factors have favoured established journals which receive the best academic output and can be very selective about what they publish. It is difficult for new journals to break into this elite league and the impact factor itself has come under attack for failing to fully capture the quality of a journal, its contents or the academics who publish in it. More recently, research funders have begun to challenge the link between impact factor and research quality and initiatives like the San Francisco Declaration on Research Assessment (DORA) have proposed alternative ways of evaluating the quality of research.

Our new impact factor is a testament to the accessibility and relevance of our articles to the academic community who can cite them, as well as the non-academic community who can grasp the gist of what we publish through our lay summaries. It vindicates our conscious decision around the diversity of our output and unconventional approach towards curating and presenting scientific thought. In addition to showcasing new discoveries, *HROpen* has published protocols, research validating previous findings, recommendations for practice and clinical guidelines.

From the start, we were committed to making science accessible to clinicians and patients across the world regardless of their familiarity with jargon or ability to pay. Our open access format has been critical in ensuring that our publications have immediate and widespread impact and are able to facilitate evidence-based decisions in healthcare. Meanwhile, the academic community has witnessed a major shift from the notion of research being an end in itself, to a recognition of the need for societal impact. Plan S, an initiative launched in September 2018 and supported by many funding organizations, mandates that from 2021, all research supported by public or private grants from research councils and other funding bodies, must be published in compliant open access journals or platforms, or made immediately available via open access repositories. Biomedical scientists are mindful of the need to make research freely available as quickly as possible so that their peers can build on the existing body of knowledge to expedite the translational pathway for new discoveries and maximize any potential health benefits.

We are grateful to ESHRE who had the vision to launch an open access online journal, our colleagues in *Human Reproduction*, *Molecular Human Reproduction* and *Human Reproduction Update* as well as Oxford University Press for all the support we have received. Most of all, we are grateful to all the authors who entrusted their work to *HROpen*, to the editors and reviewers who provided their expertise and knowledge and to our readers who have embraced our values.

As a new online journal, there have been times when some may have felt we were less visible than our more established peers. This has now changed. Our impact factor of 7.13 provides empirical proof that our articles are being read and cited. Five years into our journey, we could not have asked for more.

